# Implant Dentistry from One-Way Direction to the Reversibility of the Osseointegration

**DOI:** 10.1055/s-0041-1740219

**Published:** 2022-02-17

**Authors:** Eduardo Anitua

**Affiliations:** 1 University Institute for Regenerative Medicine and Oral Implantology- UIRMI (UPV/EHU-Fundación Eduardo Anitua), Vitoria, Spain; 2 BTI Biotechnology Institute, Vitoria, Spain

Nowadays, implant dentistry has social, economic, and clinical values. It is one of the economic motors of society. The reliability and high success rate of implant-based oral rehabilitation are fascinating, making this option predictable and safe. 1 However, dealing with an implant failure is frustrating for both clinicians and patients. For this reason, moving implant dentistry from a one-way direction to treatment reversibility would improve the management of failure cases with minimally invasive measures.

The principle of reversibility is commonly discussed in relation to the retention mechanism of the prosthesis. 2 But nowadays, in my opinion, it is extendable to the implant fixture itself (des-osseointegration). The implant size (diameter and length) and design (neck walls and connection) are factors that influence the removal torque of an implant and thus the reversibility. 3 4 5 Placing more titanium than necessary is not a virtue, and it would complicate the reversibility of implant therapy.

The reversibility (des-osseointegration) of the dental implant opens a door in the management of complications. It gives a new perspective in prosthodontic therapies that makes us better prepared to deal with the patients' needs throughout their lives. Under this perspective, the optimal position and number of dental implants are dynamic and may change as the patient's oral situation evolves, for example, the loss of additional tooth/teeth or the failure (biological/aesthetic) of the existing dental implant. The rehabilitation of improperly positioned implants is a challenge.

The reversibility (des-osseointegration) of the dental implant should be minimally invasive. 2 The instruments and gadgets that facilitate the des-osseointegration of the implant should be in line with the designs of the implants. They have the challenge of being up to date with and adapted to the implant systems on the market. Design titrations are also required to increase their effectiveness and reduce complications and risks.

The clinicians' responsibility is to apply the state of the art in case diagnosis, case selection, treatment planning, treatment execution, patient follow-up, and complications solving. Implant dentistry is a team effort; everyone involved is giving their best to the patient. The ultimate objective is to offer the patient effective and safe treatment that is in continuous adaptation to the patient's needs (aesthetic or functional). The reversibility of the osseointegration is a factor that supports the evolution of the clinical case.
